# Process Evaluation of Teaching Critical Thinking About Health Using the Informed Health Choices Intervention in Rwanda: A Mixed Methods Study

**DOI:** 10.9745/GHSP-D-23-00483

**Published:** 2024-12-20

**Authors:** Michael Mugisha, Andrew D. Oxman, Laetitia Nyirazinyoye, Anne Marie Uwitonze, Clarisse Marie Claudine Simbi, Faith Chesire, Ronald Ssenyonga, Matt Oxman, Allen Nsangi, Daniel Semakula, Margaret Kaseje, Nelson K. Sewankambo, Sarah Rosenbaum, Simon Lewin

**Affiliations:** a Institute of Health and Society, Faculty of Medicine, University of Oslo, Norway.; b School of Public Health, College of Medicine and Health Sciences, University of Rwanda, Kigali, Rwanda.; c Centre for Epidemic Interventions Research, Norwegian Institute of Public Health, Oslo, Norway.; d Tropical Institute of Community Health and Development, Kisumu, Kenya.; e Department of Medicine, Makerere University, College of Health Sciences, Kampala, Uganda.; f Faculty of Health Sciences, Oslo Metropolitan University, Oslo, Norway.; g Department of Health Sciences Ålesund, Norwegian University of Science and Technology, Norway.; h Health Systems Research Unit, South African Medical Research Council, Cape Town, South Africa.

## Abstract

**Introduction:**

We evaluated the Informed Health Choices secondary school intervention in a cluster randomized trial in Rwanda. The intervention was effective in helping students to think critically about health. In parallel to the trial, we conducted a process evaluation to assess factors affecting the implementation, impacts, and scale-up of the intervention.

**Methods:**

We used a mixed methods approach that included quantitative and qualitative methods. We collected quantitative data from teachers to evaluate the teacher training and each lesson. We conducted focus group discussions with students (n=10) and their parents/guardians (n=5). We conducted lesson observations (n=16) and key informant interviews with teachers (n=10) and school administrators (n=10) from intervention schools and policymakers (n=2). We analyzed the quantitative data using descriptive statistics. We used framework analysis and thematic content analysis to analyze the qualitative data.

**Results:**

Teachers noted that the teacher training supported their delivery of the intervention and that they made only small adaptations to fit student, teacher, or contextual needs. Students reported obtaining important skills, including recognizing health claims, understanding the need for research, and “thinking twice” before deciding. Participants saw the design of the intervention, students’ and teachers’ motivation, and school and home support as key facilitators for the implementation and impact of the intervention. Implementation barriers identified included the content of the lessons not being included in national examinations, competing priorities, and time constraints. Participants identified several factors that could facilitate intervention scale-up, including the need for the skills taught in the lessons and compatibility of the intervention with the national curriculum.

**Conclusion:**

We found that it was feasible to implement the intervention in Rwandan secondary schools and that students benefited from the intervention. Scaling up the intervention will likely require addressing the barriers identified in this study.

## Abstract (precis)

In this process evaluation, we found that teacher training, student factors, and school support helped the implementation of an intervention designed to help students think critically about health claims.

## Teaser

**Key Findings:**

- Few randomized trials have evaluated the effects of educational interventions that teach critical thinking about health. This study assessed the implementation process, impact, and scale-up of the Informed Health Choices secondary school intervention in Rwanda.
- The intervention improved students’ ability to recognize claims, assess them, and weigh the benefits and harms of interventions and helped students to use what they learned.
- The resources were compatible with the curriculum, but competing priorities and insufficient time affected the implementation and impact of the intervention and would likely affect scaling it up.

**Key Implications:**

- Teacher training is essential for effective implementation of the Informed Health Choices secondary school intervention.
- Additional time for the lessons and integrating them into the curriculum would likely increase the impact and facilitate scaling up the intervention.

See related articles by Ssenyonga et al. and Chesire et al.

## INTRODUCTION

Suggestions about what to do for our health (health claims) are ubiquitous in contemporary society. The Internet and social media have increased access to both reliable and unreliable health information.

Informed decisions about whether and how to act on health information require critical thinking—the ability to think clearly and rationally about what to do or what to believe.^1^ To cope with the bombardment of contradictory health claims that frequently originate from sources with competing interests, people need to be able to decide what to believe about the effects of health interventions and what to do.^2^ The COVID-19 infodemic (too much information, including false or misleading information) illustrated dramatically how the barrage of contradictory health information could mislead people into believing unreliable claims and not believing reliable ones.^3^

Critical thinking is a key competence in many school curricula.^4^^,^^5^ This includes East African curricula, where there is an opportunity to teach critical thinking about health, but it is not being taught currently.^6^^–^^8^ Given this need and opportunity, we developed and evaluated educational resources to enable secondary school students to think critically about health claims. We worked closely with teachers, students, curriculum specialists, policymakers, and other stakeholders to develop these resources using a human-centered design approach. We developed digital resources because the cost of scaling up their use would be low compared to printed materials, provided they could be used with available, low-cost technology.

The Informed Health Choices (IHC) secondary school resources include 10 lesson plans that focus on 9 prioritized key concepts.^9^ The IHC secondary school intervention included a teacher training workshop and use of the resources to teach the 10 lessons. We evaluated the impact of the intervention in cluster-randomized trials in Rwanda, Kenya, and Uganda.^10^^–^^12^

Process evaluations can help to explore how an intervention was implemented, its reception, and other factors affecting its effectiveness beyond the outcome measures.^13^ For example, a process evaluation linked to a randomized trial of the IHC primary school intervention in Uganda helped identify barriers to the use and scale-up of the intervention.^14^ For IHC secondary school intervention, we conducted a process evaluation alongside the randomized trial in each country.^15^^–^^17^

Process evaluations can help to explore how an intervention was implemented, its reception, and other factors affecting its effectiveness beyond the outcome measures.

We aimed to explore the implementation and impacts of the IHC secondary school intervention in Rwanda, as well as factors affecting its impact and scale-up. It addresses 3 questions: To what extent was the IHC secondary school intervention implemented as intended? What were the perceived desirable and undesirable effects of the IHC secondary school intervention? What factors could affect the impact and scale-up of the IHC secondary school intervention?

## METHODS

This was a mixed-methods process evaluation that used both qualitative and quantitative methods alongside a 2-arm, cluster-randomized trial. The process evaluation was conducted in the intervention arm of the trial (Figure). Details of the trial are reported elsewhere.^12^

**FIGURE:**
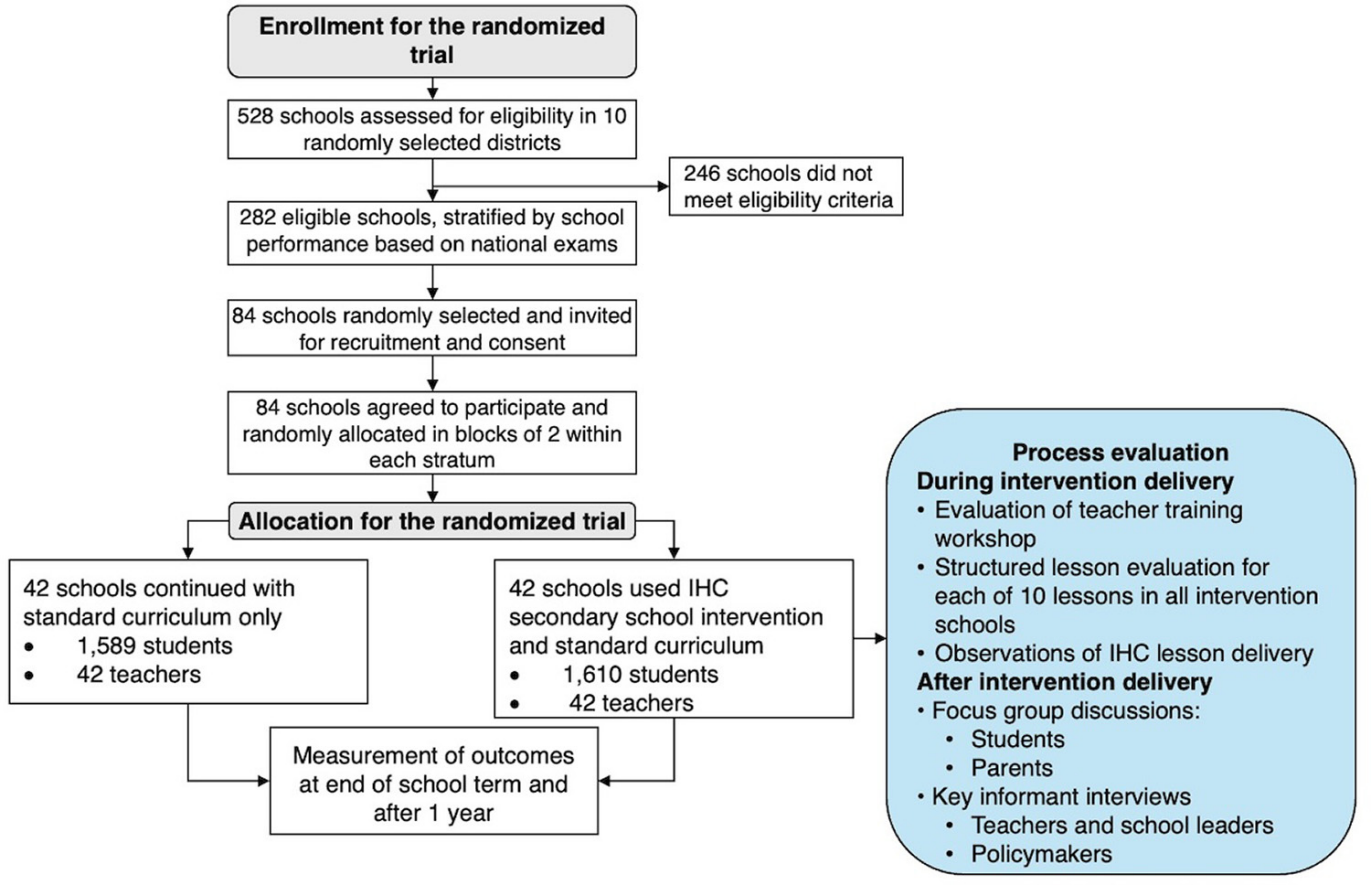
Process Evaluation Conducted as Part of the Intervention Arm of a Cluster-Randomized Trial of the Informed Health Choices Secondary School Intervention, Rwanda

### Study Setting and Location

Lower secondary schools were randomly selected to participate in the trial from 10 of 30 districts representing all 5 provinces in Rwanda. The process evaluation was conducted in schools allocated to the IHC secondary school intervention. The districts, with technical support from the Rwanda Basic Education Board, oversee school activities, staffing, and resource support. Schools are managed by the school director and 2 deputy head teachers, 1 responsible for academics and 1 for discipline. In lower secondary schools, students learn 9 core subjects, including science (mathematics, physics, chemistry, biology and health sciences, information and communication technology), languages (English, French, Kinyarwanda, and Kiswahili), and humanities (history and citizenship, geography and environment, and entrepreneurship).^18^ In lower secondary schools, students have 41 periods per week. Each period is 40 minutes. The number of periods in a specific subject depends on the weight of the subject. All subjects cover higher-order thinking skills and other key competencies, including critical thinking, creativity and innovation, research and problem-solving, communication, cooperation, interpersonal relations, life skills, and lifelong learning. On average, students are typically aged 13–15 years. The subjects are taught by teachers who have completed at least 3 years of university education or have a university degree in education. Most secondary schools have computers with Internet access, grid electricity, and projectors.

### Intervention

The educational resources included 2 versions of each lesson: 1 for classrooms with only a blackboard and 1 for classrooms with a projector. They also included a teachers’ guide and extra resources (glossary of terms, examples of claims, printouts, teacher training materials, teaching strategies, and underlying principles).^19^ The resources could be accessed online and downloaded using a smartphone or computer. The 3-day teacher training workshop was facilitated by 2 teachers who participated in the pilot of the intervention, with support from the research team. A detailed description of the intervention using the Guideline for Reporting Evidence-based practice Educational interventions and Teaching checklist is provided in Supplement 1, and a summary of that is incorporated in the Box.

BOX Summary of the Informed Health Choice Secondary School Intervention

**Goal:** To give secondary school students a basic ability to think critically about health actions (things that people do to care for their health or the health of others); understand why thinking critically is important; to be able to recognize claims about the effects of health actions; and assess some of those claims.

**Underlying theory:** We used the Informed Health Choices (IHC) key concepts framework as a starting point for this educational intervention. These concepts or principles are intended to improve people’s ability to make better decisions on what to believe and do when faced with health claims and choices. The framework is based on evidence of the importance of the included concepts, logic, feedback, and other relevant framework.

**Content and planned delivery:** The intervention included:

- 2–3-day training workshop for secondary school teachers, facilitated by other teachers, to introduce them to the IHC learning resources and learning content.
- 10 lessons for students in a single school term, with each lesson taught in 40 minutes.
- Overview and background for each of the 10 lessons for teachers. The 10 lessons were delivered by the teachers during regular classroom time or, if necessary, outside of regular classroom time. They could use a computer, smartphone, or printouts to support delivery of the lessons.

**Delivery:** Depending on what equipment was available to the teachers, teachers delivered the lessons to students using a data projector and slide presentations that are included in the digital resources. In case of a power cut, teachers were provided with a blackboard-based lessons. The number of students in a class varied. Teachers used teaching strategies such as guided note- taking, small group discussion, use of response cards, homework, use of a standard lesson structure, setting objectives, and providing feedback.

### Delivery of the Intervention

One class in each school participated in the trial. Each school in the intervention arm planned to teach the 10 lessons in 1 term (12 weeks) using their own schedule between April and July 2022. Teachers were free to adapt the teaching plan and extend the time. All the teachers used projectors and laptops to teach the lessons. They could switch to a blackboard version of a lesson when necessary. Each lesson included a review of the previous lesson, an introduction, activities, and a wrap-up. Some lessons included a homework assignment. Lessons 5 and 10 were reviews of the previous lessons.

### Logic Model

Our starting point for developing the intervention was that young people need to learn key concepts that can help them critically appraise claims about treatment effects and make informed health choices.^20^^,^^21^ We developed the educational resources^22^ to teach lower secondary school students 9 such concepts that were prioritized by teachers, curriculum specialists, and researchers from Kenya, Rwanda, and Uganda.^9^ Development of the resources was informed by context analyses in those countries,^6^^–^^8^ and an overview of systematic reviews of strategies for teaching critical thinking skills (unpublished work). Our expectation was that use of the resources would lead to a better understanding and application of those concepts as well as recognition of treatment claims, the importance of reliable comparisons of treatments by researchers, and the need to consider both advantages and disadvantages when deciding what to do. This, in turn, would increase the likelihood of students accessing and using reliable information and decrease the likelihood of their being misled by unreliable information. That would lead to students being better able to make informed health choices and, ultimately, to better health outcomes. Supplement 2 illustrates our logic model and the assumptions underlying it.

Our starting point for developing the intervention was that young people need to learn key concepts that can help them critically appraise claims about treatment effects and make informed health choices.

### Study Participants and Recruitment

We purposively selected 16 schools from the intervention arm of the trial for the process evaluation. Participants included the teachers who taught the lessons (predominantly biology and health science teachers), students in the second year of lower secondary school, and school administrators. Other participants were parents of some of those students and policymakers. In each school, we selected students purposively to encompass variation in terms of their school performance and sex. The teachers who delivered the intervention assisted us in selecting students to participate in the process evaluation. We purposively selected parents who had interacted with their children regarding the IHC intervention. The teachers asked their students whether they had interacted with their parents regarding what they learned. Through children who had interacted with their parents, we invited parents to meet our research team at their child’s school. We explained the study and invited them to participate in a group interview. We recruited the head teachers or director of studies at each of the 10 schools by visiting their schools. Through the Rwanda Basic Education Board, we recruited policymakers who had experience with the development and implementation of the IHC secondary school intervention. We interviewed them at their workplace.

### Data Collection Process

The methods, timing, sources, sampling, process, and tools used to collect data are summarized in Table 1.

**TABLE 1.** Data Collection for Process Evaluation of Informed Health Choices Secondary School Intervention, Rwanda

**Method and Timing**	**Source and Sampling**	**Data Collection Process and Tools**
Workshop evaluation to assess teachers’ perceptions of training they received (immediately after teacher training workshop)	All teachers from intervention arm who attended the training (N=42)	Teachers completed an online questionnaire with 5-point Likert response options that assessed the quality of training, extent to which training goals were achieved, and their readiness to deliver the intervention as intended.
Lesson evaluations to assess the delivery of each lesson (immediately after each lesson)	All teachers in intervention arm (N=42) for each of the 10 lessons.	Teachers completed an online lesson evaluation form describing how they prepared for and taught the lesson and the extent to which the lesson objectives were achieved.
Non-participatory observation of lessons to observe how the lessons were taught (during intervention delivery)	Intervention-arm schools (N=16) that were purposively sampled to ensure variation in ownership (private, public, or government-aided) and performance (high or low), as defined by the National Examination and School Inspection Authority. We observed all 10 lessons at least once.	We sat in classes during lesson delivery and used a structured observation form to note how the lesson was taught. We recorded how the teacher delivered the lesson and how students responded.
KIIs to explore how participants experienced the intervention (after intervention delivery)	Purposively sampled teachers (N=10) from schools that varied by type (day or boarding), ownership (private, public, or government-aided) and performance (high or low). In each school, head teachers or director of studies (N=10). Policymakers from the Rwanda Basic Education Board with experience developing and implementing the intervention (N=2).	We used semistructured interview guides to conduct the interviews. We interviewed participants at their workplace in a convenient location that also ensured privacy and quality recording of discussions. Each interview lasted for 1–1.5 hours. Two researchers conducted each interview. One person led the discussion, and another took notes and audio-recorded the discussion. We transcribed verbatim all the recordings and translated to English if the interview was conducted in Kinyarwanda.
FGDs to explore how students and their parents or caregivers experienced the intervention (after intervention delivery)	Purposively sampled schools as described above for KIIs. In each of the 10 schools, we conducted 1 focus group for students (N=10 FGDs). Students varied in terms of age, sex, and performance. Each FGD included 8–10 students. For parents, we focused on 5 of the 10 selected schools that were day schools. In those schools, we used purposive sampling to select parents who were invited to the FGDs. We invited parents who had discussed the intervention with their children. Each FGD included 8–10 parents.	We used semistructured FGD guides to conduct discussions with students and their parents, respectively. We conducted the discussions at the students’ school, in a room where no teachers or school leaders were present. For all FGDs, 1 researcher moderated, and another took notes and audio-recorded the discussion. The duration of each FGD was 1–1.5 hours. We transcribed verbatim all recordings and translated to English.

Abbreviations: FGD, focus group discussion, KII, key informant interview.

### Data Analysis

We analyzed the data in relation to each of the 3 study objectives to explore (1) to what extent was the IHC secondary school intervention implemented as intended, (2) the perceived desirable and undesirable effects of the intervention, and (3) the factors affecting the impact and scale-up of the intervention. To explore to what extent the intervention was implemented as intended, for the quantitative data, we used descriptive statistics to calculate frequencies, percentages, means, and standard deviations for data collected using the structured training and lesson evaluation forms. We summarized how teachers prepared the lessons, the number of students who attended each lesson, and the extent to which the lessons were delivered as intended.

We then analyzed all the qualitative data relevant to the first and second objectives, including free text comments from the lesson evaluations and training evaluation, nonparticipatory observations, key informant interviews, and focus group discussions (FGDs). Two of the authors (MM and AMU) did all the coding. They read all transcripts to familiarize themselves with the data, then coded all data inductively, deriving initial codes from the notes. Using a thematic analysis approach, they then summarized themes and sub-themes that emerged from the data.^23^ We used Atlas.ti software to organize and arrange data for analysis.

Lastly, we used framework analysis^24^ to address our third objective and part of the second objective. We used frameworks that we developed to explore the factors that could affect implementation, impacts and scale-up of school resources,^25^ and potential adverse effects of school resources.^26^ Framework analysis involves analyzing, classifying, and summarizing data in a framework.^23^^,^^27^

We read all notes and transcripts to familiarize ourselves with the data. Before coding using framework analysis, 1 researcher coded 2 transcripts and another 1 reviewed the codes. We discussed any disagreements, reached agreement, and came up with a shared understanding of the coding. Using the Atlas.ti software to assist with coding, we then indexed all the data using the framework, summarized data from each transcript by category, and rearranged them within and across the themes (charting). We mapped the findings from different participants and developed interpretations of these. We then summarized our findings under categories in each framework.

### Assessing Confidence in the Findings of the Process Evaluation

We assessed confidence in the main findings of our study using a version of the Confidence in the Evidence from Reviews of Qualitative research (GRADE-CERQual) approach.^28^ GRADE-CERQual is a systematic and transparent method for assessing confidence in evidence from reviews of qualitative research through the lens of 4 components: methodological limitations, data adequacy, coherence, and relevance.^29^ GRADE-CERQual was developed primarily for qualitative evidence syntheses. However, most aspects of the approach are also suitable for single studies drawing on a range of primary qualitative data sources. It was used for this purpose in our prior work.^25^^,^^30^ The same 2 authors (MM and AMU) applied the modified GRADE-CERQual approach to each finding and made a judgment about confidence in the evidence supporting each main finding. We judged confidence in the findings as being high, moderate, low, or very low. All findings started as high confidence and were downgraded if there were any important concerns for each of the 4 components.

### Reflexivity

As members of the research team, we conducted a team reflexivity process that included (1) individual written reflections on expectations of the process evaluation findings and on how their background and experience might shape the process evaluations or views of them and (2) 2 team reflexivity discussions. Key themes emerging from these reflections included issues related to the effects of the intervention, project sustainability and scaling up, the scope of the evaluation, the researchers’ relationship to the project and to the participants, and dynamics within the research team. These reflexivity discussions helped to shape our analysis and write-up of the process evaluation. We describe the team reflexivity process as well as the key themes emerging from this in more detail in Supplement 3.

### Ethical Approval

We obtained ethics approval for the trial and the process evaluation from the Rwanda National Ethics Committee (RNEC) (approval No. 691/RNEC/2019), with subsequent annual renewal and amendment in 2020 (No. 1019/RNEC/2020) and 2022 (No. 41/RNEC/2022). The approval included the consent and assent forms of the research participants. We explained to the participants the study aim, objectives, benefits, and harms that may result in participation. We obtained consent from all participants before data collection.

### Public Involvement in the Research

We engaged participants in 2 ways. First, as research participants (students, teachers, parents, and policymakers). In addition, we involved students, teachers, education and health researchers, and policymakers in the development and piloting of the IHC secondary school intervention.^31^

## RESULTS

### Characteristics of Schools Observed and Research Participants

We observed 16 lessons in 7 low-performing and 9 high-performing schools. Most were public-private owned (government-aided schools) (n=10), while others were public (n=4) or private (n=2). Each lesson in the teaching plan was observed at least once. We conducted 10 student FGDs and 5 parent FGDs. We interviewed 20 school staff (10 teachers and 10 school leaders) and 2 policymakers (Table 2).

**TABLE 2.** Characteristics of Schools and Participants in Informed Health Choices Intervention Lesson Observations, Rwanda

	**No.**
**Schools**	(N=16)
School ownership	** **
Government aided	10
Public	4
Private	2
School performance	** **
Low	7
High	9
**Participants**	
Students	(N=100)
Age, years	** **
12–15	61
16–18	39
Sex	** **
Male	42
Female	58
School staff	(N=20)
Age, years	** **
45 or younger	13
Older than 45	7
Sex	** **
Male	17
Female	3
Role	** **
Science teacher	10
School leader	10
Parents	(N=40)
Age	** **
45 or younger	17
Older than 45	23
Sex	** **
Male	10
Female	30
Policymakers	(N=2)

### Was the Intervention Implemented as Intended?

#### Teacher Training

All 42 teachers who attended the training completed the training evaluation form. Of these, 26 (61.9%) rated the training as excellent and indicated that it helped them acquire the knowledge and skills needed to deliver the intervention. Their detailed responses on how they experienced different aspects of the training and on their confidence to teach the lessons are shown in Table 3.

**TABLE 3.** Teachers’ Views of How They Experienced the Informed Health Choices Training, Rwanda

	**Agree,** **No. (%)**	**Strongly Agree,** **No. (%)**
**N=42**		
The training gave me general understanding of the critical thinking about health.	11 (26.2)	31 (73.8)
The training gave me a clear overview and flow of all lessons.	14 (33.3)	28 (66.7)
I can navigate through the website, and I know where I can find all that I need.	17 (40.5)	25 (59.5)
Now I understand all teaching strategies relevant for teaching critical thinking about health.	21 (50.0)	21 (50.0)
The training gave me teaching tips that I need to consider while teaching CHOICE lessons.	13 (30.9)	29 (69.1)
I am confident that I understand and can teach all 10 lessons.	17 (40.5)	25 (59.5)
The training met my expectations.	24 (57.1)	18 (42.9)
I will be able to apply the knowledge learned.	11 (26.2)	31 (73.8)
Overall rating of the training	16 (30.1)	26 (61.9)

#### Lesson Preparation

Teachers were required to prepare for the lesson. Teachers’ lesson evaluations indicated that they spent, on average, 30–90 minutes preparing for a lesson. In general, the time taken to prepare the lesson was longer for the first lessons and became shorter for subsequent lessons. Most teachers reported that they were prepared or very prepared to teach the lessons (Table 4). In free text comments in the lesson evaluations, they said that it was easy to prepare the lessons because the IHC resources were relevant, helpful, and provided sufficient guidance.

**TABLE 4.** Teachers’ Feedback on How Lessons Taught in the Informed Health Choices Intervention Was Delivered and How the Learning Goals Were Achieved, Rwanda

	**Lessons**									
(N=42^a^)	**1**	**2**	**3**	**4**	**5**	**6**	**7**	**8**	**9**	**10**
Lesson preparation										
Time taken to prepare the lesson (minutes), mean (SD)	50.5 (28.9)	47.4 (30.7)	47 (29.8)	44.8 (26.5)	35.5 (17.2)	44.7 (30.6)	42.2 (18)	48.6 (38.7)	42.6 (28.1)	46.4 (28.9)
Level of preparedness										
Prepared or very prepared	41 (97.6)	40 (97.6)	42 (100)	41 (97.6)	40 (100)	41 (100)	42 (100)	41 (100)	42 (100)	41 (100)
Unprepared or very unprepared	1 (2.4)	1 (2.4)	0 (0)	1 (2.4)	0 (0)	0 (0)	0 (0)	0 (0)	0 (0)	0 (0)
Lesson delivery										
Delivered on planned date										
Yes	39 (92.9)	32 (78.1)	24 (57.1)	31 (73.8)	27 (67.5)	26 (63.4)	31 (73.8)	31 (75.6)	32 (76.2)	31 (76.6)
No	3 (7.1)	9 (21.9)	18 (42.9)	11 (26.2)	13 (32.5)	15 (36.6)	11 (26.2)	10 (24.4)	10 (23.8)	10 (24.4)
Mode of delivery										
Projector based	40 (95.2)	39 (95.1)	38 (90.5)	40 (95.2)	38 (95)	38 (92.7)	39 (92.9)	41 (100)	40 (95.2)	38 (92.7)
Blackboard based	2 (4.8)	2 (4.9)	4 (9.5)	2 (4.8)	2 (5)	7 (7.3)	3 (7.1)	0(0)	2 (4.8)	3 (7.3)
Changed the mode of delivery as planned										
Yes	3 (7.1)	3 (7.3)	4 (9.5)	4 (9.5)	4 (10)	6 (14.6)	4 (9.5)	3 (7.3)	3 (7.1)	3 (7.3)
No	39 (92.9)	38 (92.7)	38 (90.5)	38 (90.5)	36 (90)	35 (85.4)	38 (90.5)	38 (92.7)	39 (92.9)	38 (92.7)
No. students who attended lesson, mean (SD)	39.9 (9.1)	39.8 (9.1)	39.2 (9.5)	39.4 (8.7)	38.5 (9.8)	39.1 (9.3)	38.8 (9.3)	38.6 (8.9)	38.6 (9.4)	38.6 (9.2)
Time taken to complete lesson, median (IQR)	46.3 (8.9)	44.1 (8.7)	44.4 (9.8)	42.3 (5.6)	42.1 (6.2)	42 (4.9)	42.4 (6.1)	41.9 (4.2)	42.3 (4.9)	43.6 (6.2)
Level of ease or difficulty in teaching the lesson										
Easy or very easy	37 (88.1)	39 (95.1)	39 (92.9)	40 (95.2)	38 (95.0)	31 (75.6)	41 (97.6)	41 (100)	41 (97.6)	39 (95.1)
Difficult	5 (11.9)	2 (4.9)	3 (7.14)	2 (4.8)	2 (5.0)	10 (24.4)	1 (2.4)	0 (0)	1 (2.4)	2 (4.9)
Extent to which lesson objectives were achieved										
Achieved or very much achieved	41 (97.6)	39 (95.1)	41 (97.6)	41 (97.6)	40 (100)	38 (92.7)	41 (97.6)	41 (100)	42 (100)	39 (95.1)
Too little achieved or unachieved	1 (2.4)	2 (4.9)	1 (2.4)	1 (2.4)	0 (0)	3 (7.3)	1 (2.4)	0 (0)	0 (0)	2 (4.9)

Abbreviations: IQR, interquartile range; SD, standard deviation.

^a^ Items where numbers do not add up to 42 had missing data.

Teachers also said that they were motivated to invest time in preparation and that their working environment (e.g., the availability of Internet, computers, and administrative support) supported this. In interviews after the intervention, teachers reported that the training, the understandability of the lessons, and careful planning were the reasons why it was easy to prepare and deliver the lessons. In addition, their experience of the earlier lessons helped them to prepare the later ones. However, some teachers noted that they struggled to get enough time to prepare the lessons during working hours due to heavy workloads and other competing activities.

#### Delivery of the Lessons and Achievement of the Lesson Goals

As shown in Table 4, most of the teachers delivered the lessons on the date planned. Most of the lessons were delivered using the projector-based version of the lessons as planned. On a few occasions, a teacher had to switch to the blackboard version, mostly due to power outages. Most teachers (38–42 of 42 teachers) felt that the lesson objectives were achieved or very much achieved.

Our lesson observations highlighted that the lessons were well attended, with few students not attending any observed lessons. The main reason for non-attendance was absence from school. We observed that teachers generally followed the lesson plan in delivering the lessons but that some teachers skipped some parts of a lesson (e.g., review of the previous lesson or the wrap-up of the lesson). We also observed that teachers were able to engage students in introductory questions and activities. Teachers and school leaders mentioned in interviews that lessons were delivered as planned due to teachers’ efforts to prepare for the lessons, stick to the lesson plans, and use the examples and questions suggested in the resources.

We observed that teachers generally followed the lesson plan in delivering the lessons but that some teachers skipped some parts of a lesson.

To some extent, teachers had to adapt the lesson to ensure that the learning goals were achieved. For lessons that teachers felt were too long, they increased the time spent if that was possible, or they rescheduled the lesson and repeated the content if there was not enough time to cover the lesson objectives. Other examples of adaptation included teachers having to look for other words to use to explain a concept or to use the local language.

In some observed lessons, teachers deviated from the lesson plan. For example, the intervention requires lesson preparation, but some teachers delivered lessons without adequate preparation. Other examples included teachers skipping the question and the main messages in the lesson wrap-up and not engaging students in class discussions.

In interviews, most of the teachers noted that students were motivated and participated actively and that this contributed to students understanding the lesson and achieving the lesson objectives. Our observations confirmed active student participation in the lessons, suggesting that they enjoyed the class and felt the content was relevant.

### What Are the Perceived Desirable and Undesirable Effects of the Intervention?

#### Understanding of the Key Concepts in the Lessons

In the student FGDs, they explained what a claim is and indicated an understanding of some weak bases for health claims, reflecting on what they learned in class. Their understanding was further illustrated by examples of claims from their daily lives. For example, most students interviewed shared at least 1 claim about an herbal medicine that is widely believed in their community. Some students who had Internet access reported that the lessons helped them to recognize how claims on social media, Google, or TV advertisements can mislead them and cited actions they could take based on information they had acquired.

Some students reported that the lessons helped them to recognize how claims on social media, Google, or TV can mislead them.

In addition, some students said that they understood concepts related to critical thinking about evidence (the need for comparisons, large studies, and random allocation). They said that for a claim to be believed, they must check whether there was research that confirmed whether the treatment works or not. However, some students could not apply the concept in an appropriate way.

*Honestly speaking, these courses were not that difficult, they only required attention. About the 7th course (large enough groups) you get to know that not all medications are good. Though they could have helped many people you do not trust them unless it was tested in large enough groups that results do not happen by chance.* —Student, private school

*The lessons were very important to us. For example, I learnt that if I want to know if a claim is reliable, I must check if the claim is supported by what the researchers found. And by doing a comparison that gives more events, you will also know if the claim is reliable or not. Most importantly, as someone who wants to be a medical doctor… if I would like to make a vaccine, I will have to choose randomly a large group of people to participate in the study, and I will use a coin to randomly create groups. And after, I will have to check the events and decide whether the vaccine is effective or not. All those procedures will also help me to know if the vaccine is harmful or not. These lessons broadened my skills and pushed me to follow my passion of becoming a medical doctor.* —Student, government-aided school

Students also indicated an understanding of the concept of weighing the benefits and harms of treatments when deciding what to do. The expression that many students used was “think twice before making a decision” when they explained the need to consider the benefits and harms of a treatment. Teachers said that some students understood the lessons based on how they contributed with relevant examples and asked relevant questions.

#### Students’ Application of Their Learning to Health Claims and Choices

Most of the students indicated that what they had learned was useful, and they showed interest in applying this to health claims and practices they encountered in their daily lives.

Although not intended by the intervention, some students said the IHC lessons led them to use health care services instead of herbal or homemade remedies. They explained they trusted health care services as reliable sources of treatments that have been researched. They also explained that skilled professionals would give reliable advice and could help them to know which condition they have and what treatment to use for that condition.

In addition, most of the students indicated that they applied what they learned by refusing to use treatments based on common claims by other students (e.g., using toothpaste to treat heartburn or using herbal medicines to treat skin rashes). Parents of students shared how the lessons influenced their children’s thinking and openness in sharing ideas about health decisions.

#### Students’ Application of Their Learning in Contexts Other Than Health

Some students reported using what they learned in contexts other than health. The concept that students transferred most easily was weighing the benefits and harms of doing something. The concept appeared to change how some students critiqued other types of choices they were making. For example, students said that the lessons helped them to think critically about personal decisions, priorities, and adherence to school rules.

*I agree with my colleague, what he said about the right choice. You may also choose bad friends, which will also cause you to be damaged. For example, you may have a friend who keeps telling you to smoke, then you will fall asleep. Then you follow, before asking yourself, “What are the harms of smoking? What are the advantages? So, you follow your friend. You should also choose a good friend.”* —Student, government-aided school

Reflecting on how students might be applying critical thinking in other settings, teachers said that students who participated in the intervention were more thoughtful, questioning, and open-minded in class. The same experience was shared by some parents who indicated their children appeared to be more open-minded after the lessons than before.

#### Teachers’ Views on How the Intervention Impacted Them

Some teachers noted that teaching the IHC lessons had influenced them in several ways. For instance, some said that the lessons helped them to apply what they taught in real life through “thinking out of the box,” not believing everything, and applying critical thinking skills.

*I had another misconception that if a person works at a health facility, whatever she is telling me is trustworthy, that you can believe it as it is her domain. They know what they are telling you, but at the end you may find that one can mislead you because you didn’t ask them anything.* — Science teacher, public school

#### Student’s Perceived Undesirable Effects

We asked students, teachers, parents, and school leaders about any disadvantages of the lessons and looked for potential unintended effects when we observed lessons. Some students experienced undesirable effects related to misapplication of what they learned in the lessons, misunderstanding of what they learned, and conflicts. These findings will be reported in detail in a qualitative evidence synthesis exploring the undesirable effects identified in this process evaluation and from the studies in Kenya and Uganda.^32^

### What Factors Affect the Impact and Scale-Up of the Intervention?

We organized factors that facilitated the impacts of the IHC secondary school intervention into 5 categories based on the framework used in the earlier IHC primary school process evaluation: the IHC educational resources, teacher-related factors, student-related factors, school-related factors, and home environment factors.^14^

#### IHC Educational Resources

Most of the students found the lessons to be interesting and easy to understand because they related to everyday life. They said that there was no need for calculations or spending sleepless nights memorizing the content. Instead, as a student said, “You could see what you learned being applied in real life.” Other students said that the materials could help change how the whole community makes decisions about their health.

A barrier was that the concepts and some terms were hard for some students to understand. They said that having the lessons in English with new and uncommon terms sometimes made it hard for them to develop a good understanding of the lesson content. They also said that they were helped by teachers who translated some words that were difficult to understand in English into their first language.

Teachers said that some of the illustrations and examples helped students to understand the lessons but agreed that some of the terms and concepts were new or hard and a bit advanced for their students. Some teachers found that some lessons were difficult to explain to their students. We observed that lesson 7 (large enough groups) was particularly hard for the teachers to teach and for the students to understand because it was a longer lesson and had a hard concept, according to the teachers.

Students, teachers, and school leaders all said that the digital format of the lessons helped and engaged students. Students said that the format encouraged them to attend and made the lessons easy to understand. Teachers said that students like subjects that use the computer laboratory more than those that just use the blackboard because they can engage more with the lesson through images and videos. However, the fact that the educational resources were digital also was viewed as a barrier by students and teachers because students could not access them outside class.

Teachers and school administrators valued the materials and thought that the lessons should be taught in other schools because they could help other students in the same age group. Teachers said that they saw the importance of the lessons because they also gained knowledge by teaching the lessons. Parents also said that the lessons helped their children and believed that the knowledge their children gained had value for their health now and in the future.

#### Teacher Factors

Teachers and school leaders said that a key factor that contributed to the effectiveness of the intervention was that teachers were motivated to deliver the lessons. Teachers said that the motivation was rooted in the value of the content and students’ interest in learning it. Teachers felt that the lessons were flexible, could be adapted to their teaching styles, were closely related to the science subjects, and facilitated discussions with students. They said that the teacher training made them confident to be able to teach the lessons effectively. This was also noted by the school leaders.

Teachers said that the motivation to deliver the lessons was rooted in the value of the content and their students’ interest in learning it.

Students felt that the teachers created room for friendly discussions about the lessons and linked what they learned in class to what they faced outside the class. They also said that teachers were able to give relevant and engaging examples.

#### Student Factors

Students’ lesson attendance was high. The students said they felt their learning needs were met, and they were motivated to learn as the lessons addressed health issues and claims that everyone experienced. They said that the lessons encouraged open discussions and helped them to use their experience.

The main barrier expressed by students was that the lessons were not taken for credit. Some students said that the fact that the lessons were not going to be considered in the end-of-term assessments demotivated them.

#### School Factors

Support from schools to teachers and students facilitated the lessons.

*We cannot teach well if school leaders did not appreciate them.* —Science teacher

School leaders provided time to teach the lessons within the school timetable and relieved teachers of some extra responsibilities to accommodate teaching the IHC lessons. School leaders also provided resources, such as access to a laptop and projector for the lessons, notebooks for students, and printing material for the teachers, when needed. However, teachers also noted that because the lessons had to be in the computer laboratory, which was needed by other classes, this hindered the intervention.

The school leaders noted that they could justify allocating time to the lessons because these addressed a cross-cutting topic (health) and developed generic competencies (critical thinking and research) that are part of the curriculum.

Teachers said that competing priorities at school and resource and time constraints were the main barriers. They noted that the IHC lessons were in the last term of the school year and came after schools had been closed for a long time due to COVID-19. Therefore, teachers were struggling to complete the curriculum. In addition, teachers commented that the lessons were an addition to their busy workload and that they struggled fitting in regular school lessons and IHC lessons. Teachers said the computer lab was needed by other classes and IHC lessons had to be prioritized over other lessons.

Another barrier was the time needed to teach the lessons. Teachers and school leaders said that 40 minutes were not enough, with some lessons taking 1 hour on average. This was confirmed in our observations.

#### Home Environment

Some students and parents said that the home environment helped learners to understand the lessons. A few students said that their parents encouraged them to learn the content of the IHC lessons because it would help them to address health challenges that they faced. In addition, they said that they discussed with parents the homework and health claims made by family members. However, some parents did not encourage their children to learn the material as they felt that lessons that did not count toward the end-of-term assessment had no value.

### Participants’ Recommendations for Scaling Up the Intervention

#### Resources Should Be Expanded to Other Areas

Teachers and school leaders expressed the need for and value of the skills taught in the lessons, noting the lessons teach health skills needed by the public. They also noted that the lessons could be applied beyond health, for example, in business, cultural, and personal decisions. They said that given the lessons’ relevance and importance, they should be taught in all primary and secondary schools. Students said that they would like to keep learning these skills in other lessons, suggesting sustaining the lessons in the school context.

#### Lessons Should Be Added to the Curriculum

Teachers and school leaders thought the IHC lessons were a good fit with the curriculum and were compatible with subjects like biology, chemistry, and entrepreneurship. They noted that the lessons should be added to the curriculum for the country, with some proposing that these could be an independent unit within biology. However, some participants thought it would be challenging to fit the lessons into the curriculum, especially because this would require waiting until it was time to review the recently implemented curriculum.

#### Lessons Should Be Extracurricular Activities

Policymakers suggested that IHC lessons should be scaled up in schools through health clubs. They said that the content of the IHC lessons should be added as topics for discussion instead. They said that the content could be scaled up through school clubs (rather than taking classroom time) because the curriculum was heavily packed. They suggested that students would benefit more if the lessons were taught as extracurricular activities, which have more time and flexibility.

#### Students Should Be Evaluated on Lessons

Both students and teachers said that making the lessons examinable would facilitate scale-up. Teachers and students felt that it could help to improve students’ understanding as well because teachers and students would give it much time as an evaluated lesson.

#### Teacher Training Should Be Expanded

School leaders, teachers, and policymakers said that IHC training should be provided to all teachers so that they could incorporate the concepts into their classes. Some teachers said that the training would help them feel responsible and prevent unnecessary conflicts between teachers and students when they tried to apply what they learned in other courses.

#### Printed Materials Should Be Readily Available

Teachers and school leaders said that it would be helpful if hard copies of books, syllabi, and handouts were provided. Teachers also agreed that they needed printed handouts in case of power failures. In addition, students said that they would like books that they could consult in the library.

#### Use Mass and Social Media to Increase Reach

Some students and teachers proposed using radio, television, and mobile applications to promote the IHC resources so that other schools could benefit from them. They said that because many people listen to the radio, it would be possible to reach even those in remote areas. Others said that YouTube could also be used for scaling up. They suggested that through social media, such as YouTube, it would be possible to reach many young people.

### Confidence in the Evidence From Reviews of Qualitative Assessment

In the CERQual assessment, we found that most of the main findings that we graded had an overall score of high confidence. The exception was the finding, “In some lessons, teachers deviated from the lesson plan mainly due to limited time.” The finding was limited to few data from lesson observations and teacher’s comments. Similarly, the finding “Teachers felt that they achieved the lesson goals for most of the lessons” was limited to the lack of coherence in how teachers reported the finding and how we observed some of the lessons. Both findings were rated moderate confidence. In addition, the finding “Policy makers, teachers and students suggested the use of school clubs, printing of IHC resources and use of social media to scale up the IHC lessons” was rated as low confidence due to lack of coherence and adequate data underlying the finding. Apart from these, all other findings were rated with high confidence, demonstrating no concerns related to methodology, relevance, coherence, and adequate data underlying the finding. The details of the CERQual assessment can be found in Supplement 4.

## DISCUSSION

The study found that teacher training was a key factor impacting the IHC secondary school intervention. Some students were able to understand the concepts taught and have applied them to health and non-health choices. Teachers have also benefited from the intervention and have found it relevant in their decisions. However, some students had misunderstandings of the content and have had conflicts with their parents and others over using what they learned. The nature of the learning resources, teacher and student motivation, school, and home enabling environments were key facilitators for the impact of IHC secondary school resources.

Barriers to the impact were competing priorities, time constraints, and that some of the terms and concepts were new or hard and a bit advanced for some students. The fact that the intervention addressed the skills needed and its compatibility with the curriculum were the main factors that could drive the scale-up of the intervention.

The findings of this process evaluation are consistent with our trial findings, although the process evaluation indicated that there was more understanding of the key concepts than the results of the multiple-choice test used to measure outcomes in the trial. The process evaluation found that at least some students understood the key concepts and applied the concepts to health decisions. The trial found that 58% of students who were in the intervention arm had a passing score on the critical thinking about health test and 23% of them mastered the concepts. Both the process evaluation and the results of the trial suggest that most teachers understood the concepts—all but 1 teacher had a passing score and 76% had a mastery score.

The process evaluation found that at least some students understood the key concepts and applied the concepts to health decisions.

Both the process evaluation and the trial indicate that the intervention was helpful for both low- and high-performing students. The process evaluation found that it was hard for some students to understand some of the English vocabulary used in the resources and some of the concepts. Similarly, metanalysis of the 3 trials in Kenya, Rwanda, and Uganda found that the intervention was less effective in students who lacked English proficiency compared to students with advanced proficiency.

The process evaluation of the IHC primary school intervention in Uganda found similar facilitators and barriers for the implementation and impact of the intervention to what we found in this study.^25^ However, our study found that students started using what they learned in daily life. This may have occurred less in the primary school intervention due to differences in the age of participants.

The IHC secondary school resources were developed using human-centered design with engagement of students, teachers, and curriculum specialists in Rwanda, Kenya, and Uganda.^22^ The goal was to ensure that the intervention was fit for the intended users and context. Our process evaluation findings indicate that the intervention was valued and found useful by students and teachers and that this facilitated the impact of the intervention. In addition, context analyses in Rwanda, Kenya, and Uganda informed the design of the intervention.^6^^–^^8^ These analyses indicated the compatibility of the concepts in the lessons with the curricula in Kenya, Rwanda, and Uganda. The findings of this process evaluation confirm this.

When designing the intervention, we attempted to address most of the challenges identified in the context analysis. However, we were unable to address some barriers, such as the content not being examinable, competing priorities, and insufficient time for the lessons. Nonetheless, students, teachers, parents, and education authorities believe that students learned the necessary skills for making informed health choices and that they started to use these skills.

### Potential for Sustainability and Scalability

Scaling up the intervention will require integrating the lessons into the curriculum. Health, research, and critical thinking are cross-cutting themes in the Rwandan curriculum. Integrating the IHC lessons may require teaching the lessons across subjects and connecting them to different subjects, as comprehensive sexuality education was incorporated into the curriculum. However, an already packed curriculum will remain a barrier. Other approaches, such as incorporating the lessons into extracurricular activities, may, therefore, be necessary, at least initially. A disadvantage of this would be that only students who chose to participate in those activities would benefit and they still might not prioritize spending time on IHC lessons over spending time on examinable subjects or other activities such as sports.

Nonetheless, the project attempted to address the sustainability and scalability of the intervention in several ways. First, because the designed intervention was digital, it facilitated scale-up at a relatively low cost. Digital resources potentially reduce the substantial costs that would be required with paper-based resources. In addition, in designing the intervention, we involved key stakeholders, including the curriculum department, students, and teachers, to ensure the intervention would be relevant to the target groups. Furthermore, we held a webinar with representatives from the curriculum and education offices of 3 East African countries to discuss scalability and opportunities to incorporate the intervention into the national curriculum, along with what remains to be done to gather more evidence around this intervention. We obtained commitments of support from the 3 countries, and we continue engaging these officials.

### Strength and Limitations

The strengths of this study include describing our methods in a published study protocol,^17^ including data from multiple sources and forms of data collection, and triangulating the findings. More than 1 member of the research team coded, charted, and interpreted the findings, and we assessed confidence in our findings using an adapted GRADE-CERQual.

A limitation may be that we used a framework for barriers and facilitators developed by our team.^14^ That may have affected our observations and analyses. We mitigated this risk by keeping ourselves open to other new ideas and analyzing data using both framework analysis and thematic content analysis. Another possible limitation is that we both developed and evaluated the intervention. This may have led us to overreport and interpret positive findings and to underreport and interpret negative findings. However, we ensured that 1 of the authors (AMU) who participated in the analysis was not involved in the development of the intervention and data collection, in addition to using GRADE-CERQual to assess confidence in the findings in a transparent way.

## CONCLUSION

The study found that the IHC secondary school intervention was implemented as intended with minimal adaptation to fit the needs of teachers, students, or contexts. The findings of the process evaluation are consistent with those of the trial. Some students acquired important skills that they started to use for health choices as well as choices in other areas of their lives. Participants found the intervention valuable and useful. The facilitators and barriers that we identified will inform the development of plans to scale up the intervention in Rwanda.

Future research should focus on exploring ways to scale up the intervention, integrate it into the curriculum, add more concepts and lessons, and understand what it takes to institutionalize working interventions that would benefit students. It should also focus on developing resources that target other groups of people, including patients, parents, and health professionals.

## Supplementary Material

GHSP-D-23-00483-Supplement.pdf

## Notes

**Peer Reviewed**
