# Supplementary material for: Process Evaluation of Teaching Critical Thinking About Health Using the Informed Health Choices Intervention in Rwanda: A Mixed Methods Study
Source: Glob Health Sci Pract. 2024 Dec 20;12(6):e2300483. doi: 10.9745/GHSP-D-23-00483 (PMC11666086; doi:10.9745/GHSP-D-23-00483)
Supplement: GHSP-D-23-00483-Supplement.pdf [file GHSP-D-23-00483-Supplement.pdf]

Supplement to: Mugisha M, Oxman AD, Nyirazinyoye L, et al. Process evaluation of teaching critical thinking about health using the Informed Health Choices intervention in Rwanda: a mixed methods study. *Glob Health Sci Pract*. 2024;12(6):e2300483. https://doi.org/10.9745/GHSP-D-23-00483

SUPPLEMENT 1. GREET checklist.

Guideline for reporting evidence-based practice educational interventions and teaching (GREET) checklist[1]

BRIEF NAME

1. **Intervention:** Informed Health Choices (IHC) secondary school intervention

The intervention was compared to routine practice (teaching according to the national lower-secondary school curriculum without intervening).

WHY this educational process

2. **Theory:** The IHC secondary school resources are based on the *IHC Key Concepts* framework. The framework includes concepts (principles) that people should understand and apply when deciding whether to believe a claim about the effects of health actions (things that people do to care for their health or the health of others) and what to do.[2,3] The framework is based on evidence of the importance of the included concepts,[4,5] logic, feedback, other relevant frameworks, [6] and adaptation of the IHC Key Concepts to other types of interventions such as educational, environmental, and policing interventions.[7]

The resources were developed by the investigators between 2020 and 2022 using human-centred design methods.[8] This included cycles of idea generation and prototyping, piloting with observation, user-testing with teachers and students, and feedback from teachers, students, and curriculum developers in Kenya, Rwanda, and Uganda, and an international advisory group. The aim of the design process was to ensure that teachers and students find the resources to be engaging, useful, and easy to use.

The teaching strategies used in the resources were based in part on an overview of systematic reviews of teaching strategies, [9] and draw on several educational theories. These include social constructivist theory (which postulates that learning can be maximized through well-designed, intentional social interaction with other learners), [10] the theory of active student response (which postulates that learning is enhanced by high levels of active student response), [11] and the elaborative retrieval hypothesis (which postulates that the search for correct answers on practice tests or quizzes results in multiple retrieval routes which aid later recall). [12]

3. **Learning objectives:** The primary learning goal is for students to have a basic ability to think critically about health actions and understand why this is important. They should be able to recognise claims about the effects of health actions and assess some of those claims. They should understand why it is important for them that researchers study the effects of health actions and recognise two key features of reliable comparisons of health actions. They should recognise that

health actions can have both advantages and disadvantages and the importance of weighing the benefits and savings against the harms and costs when deciding what to do.

4. **Evidence-based practice content:** The resources focus on nine IHC Key Concepts that were prioritised by curriculum developers, teachers, and researchers in Kenya, Rwanda, and Uganda.[13]

WHAT

5. **Materials:** The IHC secondary school resources (*Be smart about your health*) are open access digital resources for lower-secondary school teachers. The 10 lessons are provided as lesson plans in two formats: for teachers who are using either a blackboard and or a projector in the classroom. The aim is for students to learn to think critically about health claims and choices. The resources were made available to schools in the intervention group. Teachers in those schools downloaded the resources to a computer or smartphone and delivered the lessons. Schools in both the control and intervention group continued teaching the national curriculum, which did not include teaching critical thinking about health. No additional materials were provided to the control schools.

Each Lesson includes an introduction, an activity, and a wrap-up. The introduction includes the key messages from the previous lesson, a question about the previous lesson, and what this lesson is about. The activity is designed to help students achieve the learning goals. The wrap-up includes a question about what was learned, the key messages for the lesson, a homework assignment, if there is one, and what the next lesson is about. Lessons 5 and 10 include quizzes and discussions of application of what students learned in their daily lives.

For each of the 10 lessons there is an overview and background for teachers. The overview includes learning goals, key terms introduced in the lesson, and the main teaching strategies used in the lesson. The background includes a description of what the lesson is about and if relevant, common misunderstandings and closely related content that is not covered in the lesson.

In addition, there is a teachers' guide, materials for teacher training workshops, information about how to use the resources (help), optional printouts (PDFs) for teachers and students, and a glossary. Teachers were provided with binders with printouts at the training workshops.

6. **Educational strategies:** Key strategies used across lessons included guided note taking, small group discussion, use of response cards,[11] homework, use of a standard lesson structure, setting objectives and providing feedback, and multimedia design. Other strategies used in some of the lessons include concept cartoons, inquiry-based instruction, and role play.

7. **Incentives:** The incentive for teachers and students was the value they perceived in learning to think critically about health actions. Teachers at schools without Internet access were reimbursed

for the cost of downloading the resources and any other costs related to participation in the trial. They were not paid for participating in the trial and there were no other financial incentives for the schools, head teachers, teachers, or students. The evaluation administered at the end of the school term did not count towards the students' school marks or assessment of the teachers or schools.

WHO PROVIDED

8. **Instructors**: The head teacher at each participating school selected a teacher of a relevant subject (e.g., biology) for year-1 or year-2 of lower-secondary school. The teachers were invited to a 2-3-day workshop to introduce them to the resources and the learning content. The training was facilitated by other teachers who had participated in one of the teacher networks that helped to develop the resources or who piloted use of the resources. The facilitators were provided with presentations and other materials for the workshops, and they reviewed the material and plans for the workshops with the research teams prior to the workshops.

HOW

9. **Delivery:** The 10 lessons were delivered by the teachers during regular classroom time or, if necessary, outside of regular classroom time. They could use a computer, smartphone, or printouts when delivering the lessons. Depending on what equipment was available to the teachers, they delivered the lessons to students using only a blackboard or using a projector and slide presentations that are included in the digital resources. The number of students in a class varied.

WHERE

10. **Environment:** Representative samples of schools were recruited, including rural and urban schools. The conditions in the schools varied. Details of the context can be found in report of the context analysis undertaken prior to developing the resources. [14]

WHEN and HOW MUCH

11. **Schedule:** The 10 lessons were taught in a single school term. Each school decided how to fit the lessons into the schedule for that term.

12. **Amount of time**: Each lesson was designed to be delivered in a single period (40 minutes). The students were encouraged to collect and assess claims about the effects of health actions

outside of class and to discuss claims with their families and friends. The teachers needed up to 30 minutes to prepare for each lesson.

PLANNED CHANGES

13. **Adaptation**: No specific adaptation was required, but teachers were able to adapt the lessons, for example by using different or additional examples or editing the presentations.

UNPLANNED CHANGES

14. **Modifications**: As part of the process evaluations, teachers were asked to complete an evaluation form after each lesson, including information about changes they made to the lesson plan, and some teachers were observed for one to two lessons. No feedback was given to the teachers during the trial.

HOW WELL

15. **Attendance:** The teachers were asked to record attendance for each lesson. Students were encouraged to attend all lessons by telling them when the next lesson would be and its learning goals. The lessons were designed to appeal to students and to make clear the relevance and importance of the learning goals.

16. **Fidelity**: We will explore the extent to which the lessons were delivered as planned in the process evaluation, based on the evaluation forms completed by teachers after each lesson, observations of their teaching a lesson, and interviews with teachers and students.

17. **Delivery schedule**: The teachers were asked to record when each lesson was taught, the duration of each lesson, and whether all the lesson were completed as planned.

References

1. Phillips AC, Lewis LK, McEvoy MP, et al. Development and validation of the guideline for reporting evidence-based practice educational interventions and teaching (GREET). *BMC Med Educ* 2016; **16**: 237.
2. Oxman AD, Chalmers I, Austvoll-Dahlgren A, Informed Health Choices Group. Key Concepts for assessing claims about treatment effects and making well-informed treatment choices. *F1000Res* 2019; **7**: 1784.
3. Oxman AD, Chalmers I, Dahlgren A, Informed Health Choices Group. Key Concepts for assessing claims about treatment effects and making well-informed treatment choices. Version 2019. *IHC Working Paper* 2019.

4. Oxman AD, Chalmers I, Dahlgren A, Informed Health Choices Group. Key Concepts for Informed Health Choices: a framework for enabling people to think critically about health claims (Version 2022). *IHC Working Paper* 2022.
5. Oxman AD, Chalmers I, Dahlgren A. Key Concepts for Informed Health Choices: Where's the evidence? *F1000Res* 2022; **11**: 890.
6. Oxman AD, Martinez Garcia L. Comparison of the Informed Health Choices Key Concepts to other frameworks that are relevant to learning how to think critically about treatment claims, comparisons, and choices: protocol for a mapping review. *IHC Working Paper* 2018.
7. Aronson JK, Barends E, Boruch R, et al. Key concepts for making informed choices. *Nature* 2019; **572**(7769): 303-6.
8. Rosenbaum SE, Moberg J, Chesire F, et al. Teaching critical thinking about health information and choices in secondary schools: human-centred design of digital resources. *F1000Res* 2023.
9. Oxman AD, Nsangi A, Martinez Garcia L, et al. The effects of teaching strategies on learning to think critically in primary and secondary schools: an overview of systematic reviews. *Review of Education* Submitted 20 January 2023.
10. Igel C. Cooperative learning. Denver, CO: McREL International, 2010.
11. Randolph JJ. Meta-analysis of the research on response cards: effects on test achievement, quiz achievement, participation, and off-task behavior. *J Posit Behav Interv* 2007; **9**(2): 113-28.
12. Pan SC, Rickard TC. Transfer of test-enhanced learning: meta-analytic review and synthesis. *Psychol Bull* 2018; **144**(7): 710-56.
13. Agaba JJ, Chesire F, Mugisha M, et al. Prioritisation of Informed Health Choices (IHC) Key Concepts to be included in lower-secondary school resources: a consensus study. *PLoS One* 2023; **18**: e0267422.
14. Mugisha M, Uwitonze AM, Chesire F, et al. Teaching critical thinking about health using digital technology in lower secondary schools in Rwanda: A qualitative context analysis. *PLoS One* 2021; **16**(3): e0248773.

Supplement to: Mugisha M, Oxman AD, Nyirazinyoye L, et al. Process evaluation of teaching critical thinking about health using the Informed Health Choices intervention in Rwanda: a mixed methods study. *Glob Health Sci Pract*. 2024;12(6):e2300483. https://doi.org/10.9745/GHSP-D-23-00483

SUPPLEMENT 2. Logic model of the implementation of the IHC secondary school intervention in Rwanda

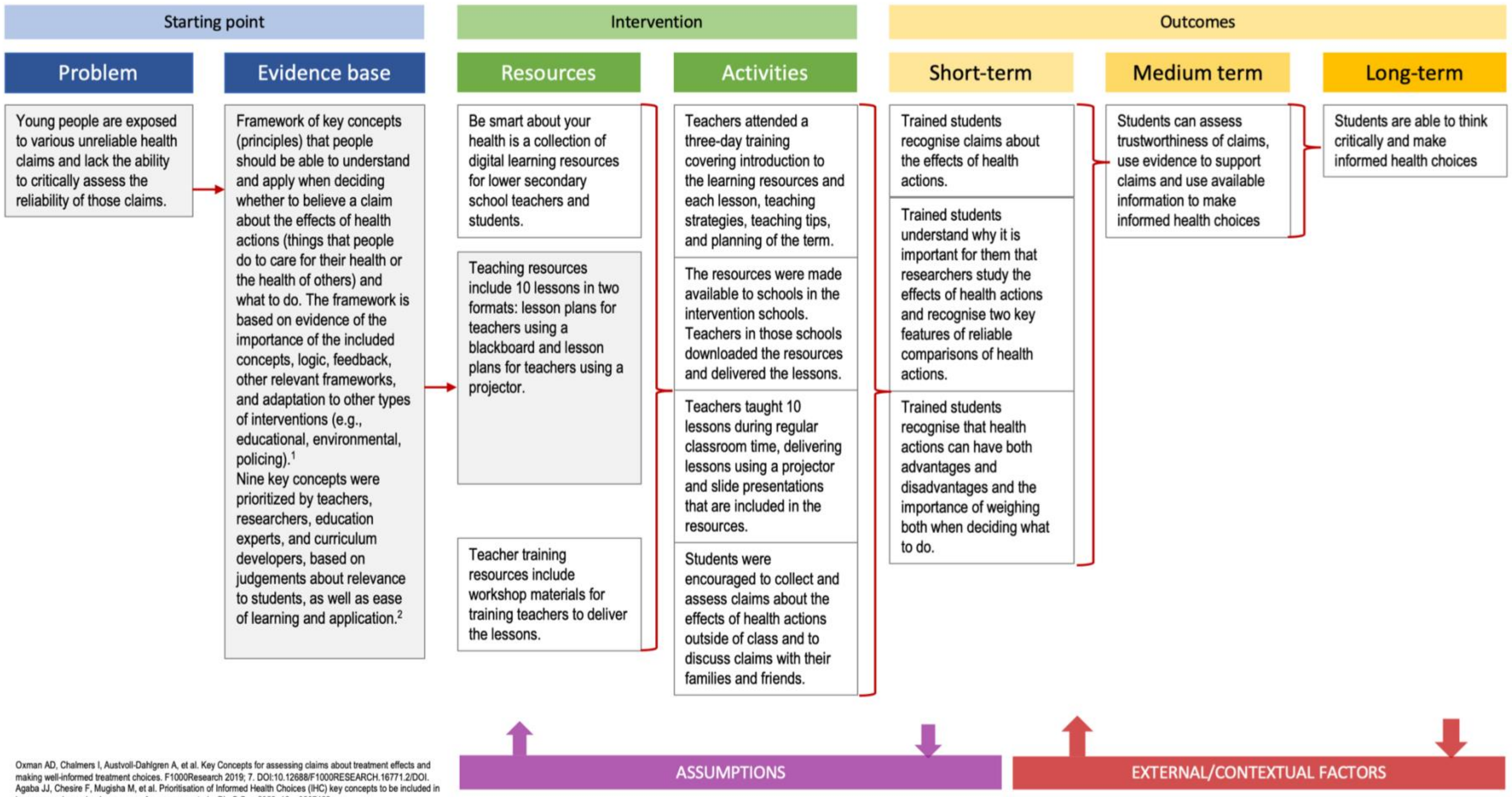

1. Oxman AD, Chalmers I, Austvoll-Dahlgren A, et al. Key Concepts for assessing claims about treatment effects and making well-informed treatment choices. F1000Research 2019; 7. DOI:10.12688/F1000RESEARCH.16771.2/DOI.
2. Agaba JJ, Chesire F, Mugisha M, et al. Prioritisation of Informed Health Choices (IHC) key concepts to be included in lower secondary school resources: A consensus study. PLoS One 2023; 18: e0267422.

Table 2. Logic model assumptions and external factors affecting the intervention.

ASSUMPTIONS	EXTERNAL/CONTEXTUAL FACTORS
1. Lessons developed are useful and fit for the context.<br>2. Teacher training enables them to deliver the intervention as intended.<br>3. Teachers can teach lessons with support of the lesson plan.<br>4. Students participate fully in learning the resources.<br>5. Schools are willing to dedicate time and resources for learning the content.	1. ICT factors (computers, projectors, internet) and their use for teaching and learning<br>2. School administration support (avail class, dedicate time, support teacher in lesson delivery)<br>3. National curriculum and demand for the teaching critical thinking skills<br>4. Educational leaders at the district and national level support the intervention delivery.<br>5. Parents and home members discuss the content of the intervention with students.

SUPPLEMENT 3. Team reflexivity considerations

By considering and communicating reflexivity considerations, researchers are able to explore and express how their *a priori* values, views, experiences and beliefs about the topic of interest, as well as their context, may influence the research being undertaken (1, 2). While reflexivity considerations are often undertaken individually, there are multiple benefits to also considering how the dynamics, structure and expectations of the research team may influence the research (3). In a team reflexivity process, all relevant members of the research team can discuss how their individual and collective perspectives, beliefs and experiences could have influenced the design and or conduct of the process evaluation, and/or their interpretation of the findings.

Team reflexivity process methods

Written reflections from the team

All members of the research team (FC, RS, SL, AN, DS, SR, JM, AO, AF, NS, MM, MO, MK, AF) were asked to write their responses to the following questions:

- What findings do I expect to come out of the process evaluations?
- How do I anticipate that the findings will contribute to the CHOICE project overall?
- How might I shape the process evaluations or my views of them, based on my beliefs (e.g., about the impacts of the intervention); background and previous research experiences (e.g., my disciplinary training); or hopes or concerns related to the CHOICE project?
- What are my concerns related to the CHOICE project, if any?

The written responses were coded and analyzed using thematic analysis methods by one member of the research team (AN).

Team discussions

We conducted two team discussions with all members of the research team (AF, CH, MK, SL, JM, MM, AN, AO, MO, SR, DS, NS). The team reflexivity discussions took place in January and April 2023. This was after data collection for the process evaluations took place, but before the team started data analysis. Each discussion lasted one to two hours. We used findings from the analysis of the written reflections to guide the discussion. The first team discussion focused on the first three questions in the list above. Based on the topics covered in the first team discussion, we organized the second discussion around the following themes:

- What are other concerns aside from implementation and sustainability (covered during first discussion)?
- Where do these concerns come from?
- What are the relationships between the project team members and how does that impact the research?

Parts of the first team discussion were recorded. The second team discussion was recorded, but the recording was destroyed before transcription. Two people observed and took notes for each discussion.

Analysing our reflections

AN used thematic analysis to identify themes from the written reflections shared by research team members. HMK used framework analysis to identify themes from the team discussions. HMK used thematic analysis to combine the analyses from the written reflections and the group discussions.

Team reflexivity considerations

Ten members of the research team submitted written reflections. 15 researchers participated in the first team discussion and ten researchers participated in the second team discussion. The main reflexivity considerations that emerged were: expectations of the findings from the process evaluations, concerns related to the CHOICE project, and team dynamics.

Background of researchers

The research team consists of 16 researchers who represent a wide array of methodological experience, experience with Informed Health Choices educational resources, and geographic and cultural backgrounds. Most of the researchers (SL, AN, DS, SR, JM, AO, AF, NS, MM, MO, MK, AF) were involved in the development of Informed Health Choices educational resources in an earlier project exploring the effect of these resources among to teach primary school children to assess claims about treatment effects (4). Four of the researchers are leading various components of the CHOICE project as part of their doctoral work. None of the researchers are teachers, educational specialists, curriculum developers, or otherwise involved with the development or implementation of school curricula.

Expectations regarding the process evaluation findings

All members of the research team indicated that they expected findings from the process evaluation to be mostly positive (e.g., students and teachers using what they learned and viewing the learning resources as being well-structured and suitable for student; the resources being relevant for daily life; and leading to improvements in teachers' skills). In addition, some researchers expected there to be clear examples of students and teachers applying what they learned in real life settings (called transfer or far transfer by team members). However, all members of the team also noted (to varying degrees) that they expected important challenges to emerge from the process evaluation (e.g., lack of time, teachers not feeling prepared, infrastructure issues, supervision gaps, resource constraints).

The researchers were almost unanimous in their view that the findings from the process evaluations would inform decisions about scaling up implementation of the intervention, and also inform future research on developing and evaluating the learning resources. Other team members also mentioned that they hoped the findings could be used to apply for future funding to continue development and evaluation of IHC resources.

Reflections on the process evaluation

Given the variety of backgrounds represented in this research project, and different perspectives on how to interpret the emerging process evaluation findings, it is perhaps unsurprising that different concerns regarding the project emerged. The concerns can be divided into the following categories: effects of intervention, project sustainability, wider perspective, and the researchers' relationship to participants.

Considerations regarding the effects of the intervention

Some team members were concerned that a substantial proportion of children receiving the intervention did poorly on the evaluation tests used in the trial, and that both the trial and process evaluation findings suggested that there were some misunderstandings of the key concepts among students and teachers. Others mentioned the potential disadvantages of measuring treatment-inherent outcomes and only near transfer (rather than far transfer). Some team members raised concerns that the research team was not focused enough on assessing the real-world importance of the benefits or how to interpret the effects of the intervention (e.g., what does it mean that students pass a test on key concepts?). Furthermore, many team members expressed concerns about the challenges of assessing the impacts of the resources on decision making in participants' daily lives (transfer).

Considerations regarding the project sustainability and scaling up

Some team members discussed the sustainability of the intervention and issues related to how to scale up implementation of the resources to other settings. Team members' views fell broadly into two groups: firstly, concerns about how to scale up the project and identify innovative methods to improve uptake of the educational resources outside of a research context. Secondly, a view that the team should take a step back and consider whether the project should be scaled up at all. The latter opinion was informed by uncertainties regarding the benefits of the intervention for the day-to-day lives of students, and whether it is worth investing resources in scaling up.

Considerations regarding the scope of the evaluation

Some members of the research team were concerned that the research team has potentially viewed the findings in a limited way (i.e., only within the scope of the project) and has not sufficiently explored how this project fits in with, or could be enhanced by, other research in the field. However, others noted that the project used a very practical approach and focused on identifying issues that could be addressed and improved upon in further research.

Considerations regarding the researchers' relationship to the project and to the participants

Two members were concerned that the research team was both responsible for implementing the intervention and undertaking the process evaluation. They noted that this could have hindered honest and/or critical feedback from the research participants (e.g., teachers).

Considerations regarding dynamics within the research team

The research team had considerable discussion about team dynamics, particularly about the researchers' backgrounds and roles in the project, and hierarchy.

The team generally saw it as advantageous that researchers from contexts of the evaluations were responsible for implementing the research in those contexts. They thought that the design of the educational resources and the conduct of the studies had been improved by input from researchers with an in-depth understanding of each context.

Some members of the team expressed concerns that the team consisted mostly of individuals with health-related backgrounds. It may have been advantageous to have included researchers with educational research and curriculum development when designing the study and interpreting the findings. In the discussions, it was noted that the research team attempted to mitigate this potential weakness by involving stakeholders (e.g., teachers, curriculum developers) at all stages of the research process.

Some members of the team also highlighted that the roles of particular researchers in the project may influence their interpretation of the findings from the trial and the process evaluation. They suggested that those members of the team who had been involved in developing and evaluating the IHC educational resources in a previous project may lean toward over overstating positive findings and may pay less attention to potential harms or negative findings. In contrast, those who have responsibility for exploring adverse effects may focus on negative findings and downplay positive findings.

The role of the researcher also emerged as a theme during discussions of team dynamics. Some of the more junior researchers noted that having a very senior researcher leading the project could have been a barrier to sharing critical opinions. However, they felt that in this project there were opportunities for sharing opinions freely, including disagreeing about methods and interpretation, and a general openness.

Finally, given that the study took place largely during the COVID-19 pandemic, there were few opportunities in the earlier phases of the project for face-to-face meetings or team-building events. Some team members noted that this may have impacted on the way in which the team worked together. During the last phase of the project (data analysis and planning the 1-year follow up) many of the project team members met regularly face-to-face.

References

1. Downe S, Finlayson KW, Lawrie TA, Lewin SA, Glenton C, Rosenbaum S, et al. Qualitative evidence synthesis (QES) for guidelines: paper 1–Using qualitative evidence synthesis to inform guideline scope and develop qualitative findings statements. Health research policy and systems. 2019;17(1):1-12.
2. Olmos-Vega FM, Stalmeijer RE, Varpio L, Kahlke R. A practical guide to reflexivity in qualitative research: AMEE Guide No. 149. Medical teacher. 2022:1-11.
3. Barry CA, Britten N, Barber N, Bradley C, Stevenson F. Using reflexivity to optimize teamwork in qualitative research. Qualitative health research. 1999;9(1):26-44.
4. Nsangi A, Semakula D, Rosenbaum SE, Oxman AD, Oxman M, Morelli A, et al. Development of the informed health choices resources in four countries to teach primary school children to assess claims about treatment effects: a qualitative study employing a user-centred approach. Pilot and Feasibility Studies. 2020;6(1):1-15.

SUPPLEMENT 4. CERQUAL ASSESSMENT OF THE MAIN FINDING

No	**Main findings**	**Methods and data sources underlying a finding**	**Overall CERQual assessment**	**Comment on the study finding**
	**Implementation of the intervention**			
1	Most of the teachers agreed that the teacher training was essential and gave them knowledge and skills to deliver the intervention as planned.	Key informant interviews, lesson and training evaluation forms	High	No concerns related to methodology, relevance, coherence and adequate of data underlying the finding.
2	Some teachers said that they took time to prepare and plan well to deliver the intervention.	Key informant interviews, lesson observations, lesson evaluation forms	High	No concerns related to methodology, relevance, coherence and adequate of data underlying the finding.
3	Most teachers said that there was not enough time to prepare due to competing activities.	Key informant interviews, lesson observations, lesson evaluation forms	High	No concerns related to methodology, relevance, coherence and adequate of data underlying the finding.
4	All schools taught all the lessons and most of the students attended the lessons with few absent.	Lesson evaluation forms, lesson observations, key informant interviews	High	No concerns related to methodology, relevance, coherence and adequate of data underlying the finding.
5	Most of the teachers followed the lesson plans with minimal adaptation.	Lesson evaluation forms, lesson observations, key informant interviews	High	No concerns related to methodology, relevance, coherence and adequate of data underlying the finding.
6	For some lessons that teachers felt were too long, they increased the time spent if that was possible, or they rescheduled the lesson and repeat the content if there was not enough time to cover the lesson objectives	Lesson observations, key informant interviews	Moderate	The study finding is limited to our few observations and some reports from teachers.

No	Main findings	Methods and data sources underlying a finding	Overall CERQual assessment	Comment on the study finding
7	Most of the teachers noted that students were motivated and participated actively, and that this contributed to students understanding the lesson and achieving the lesson objectives.	Lesson evaluation forms, key informant interviews, lesson observations	Moderate	There were concerns regarding coherence of data underlying a finding. Although teachers reported having achieved lesson gioals, Some of our observations indicated that some lesson goals were not met.
	**Perceived desirable effects of the intervention**			
	**Understanding of the key concepts**			
8	Students explained what a claim is and indicated an understanding of some weak bases for health claims, reflecting on what they learnt in class. Their understanding was further illustrated by examples of claims from their daily lives.	Focus group discussions, lesson observations	High	No concerns related to methodology, relevance, coherence and adequate of data underlying the finding.
9	Students said that they understood concepts related to critical thinking about evidence (the need for comparisons, for large studies, and for random allocation) but some could not apply the concept in an appropriate way.	Focus group discussions, lesson observations	High	No concerns related to methodology, relevance, coherence and adequate of data underlying the finding.
10	Students also indicated an understanding of the concept of weighing the benefits and harms of treatments when deciding what to do.	Focus group discussions swith students, lesson observations	High	No concerns related to methodology, relevance, coherence and adequate of data underlying the finding.
	**Application of lessons in health context**			

No	**Main findings**	**Methods and data sources underlying a finding**	**Overall CERQual assessment**	**Comment on the study finding**
11	Students indicated that what they had learnt was useful, and they showed interest in applying this to health claims and practices they encountered in their day-to-day lives	Focus group discussions with students and parents	High	No concerns related to methodology, relevance, coherence and adequate of data underlying the finding.
	**Use of what learned in other decisions/choices**			
12	Some students reported using what they learned in contexts other than health. The concept that students transferred most easily was weighing the benefits and harms of doing something.	Focus group discussions with students and parents	High	No concerns related to methodology, relevance, coherence and adequate of data underlying the finding.
13	Teachers and parents said that students who participated in the intervention arm more thoughtful, questioning, and open minded.	Key informant interviews with teachers, focus group discussions with parents	High	No concerns related to methodology, relevance, coherence and adequate of data underlying the finding.
	**Teachers views on how the intervention impacted them**			
14	Some teachers said that these lessons helped them to apply what they taught in real life through ‘thinking out of the box’, not believing everything, and applying critical thinking skills.	Key informant interviews with teachers	High	No concerns related to methodology, relevance, coherence and adequate of data underlying the finding.
	**Factors facilitating impact of the intervention**			
15	Most of students found the lessons to be interesting and easy to understand because these related to everyday life.	Focus group discussions with students.	High	No concerns related to methodology, relevance, coherence and adequate of data underlying the finding.

No	**Main findings**	**Methods and data sources underlying a finding**	**Overall CERQual assessment**	**Comment on the study finding**
16	Students, teachers, and school leaders all said that the digital format of the lessons helped and engaged students.	Key informant interviews with teachers and school administrators, focus group discussions with students	High	No concerns related to methodology, relevance, coherence and adequate of data underlying the finding.
17	Students felt that the teachers created room for friendly discussions about the lessons and linked what they learned in class to what they faced outside the class.	Focus group discussions with students.	High	No concerns related to methodology, relevance, coherence and adequate of data underlying the finding.
18	Techers and school leaders said that a key factor that contributed to the effectiveness of the intervention was that teachers were motivated to deliver the lessons.	Key informant interviews with teachers and school administrators, focus group discussions with students	High	No concerns related to methodology, relevance, coherence and adequate of data underlying the finding.
19	Teachers and students found the lessons easy to understand. Teachers, students, and parents found them useful. Teachers and school administrators valued the lessons.	Key informant interviews with teachers and school administrators, focus group discussions with students and with parents	High	No concerns related to methodology, relevance, coherence and adequate of data underlying the finding.
20	Students were motivated to learn and attended the lessons.	Key informant interviews with teachers, focus group discussions with students, lesson observations, and lesson evaluation forms	High	No concerns related to methodology, relevance, coherence and adequate of data underlying the finding
21	School administration and management supported the delivery of the lessons by providing the necessary resources and time.	Key informant interviews with teachers and school leaders	High	No concerns related to methodology, relevance, coherence and adequate of data underlying the finding

No	Main findings	Methods and data sources underlying a finding	Overall CERQual assessment	Comment on the study finding
22	Parents' support and home environments helped students to understand the lessons.	Focus group discussions with parents and students	High	No concerns related to methodology, relevance, coherence and adequate of data underlying the finding
	**Barriers to effective delivery**			
23	A barrier related to the lessons was that the concepts and some terms were hard for the students to understand.	Focus group discussions with students	High	No concerns related to methodology, relevance, coherence and adequate of data underlying the finding
24	The fact that the educational resources were digital was viewed as a barrier by students and teachers because students could not access them outside class.	Key informant interviews with teachers, focus group discussions with students	High	No concerns related to methodology, relevance, coherence and adequate of data underlying the finding
25	Some of the concepts taught in the lessons were difficult and some lessons were hard to understand.	Key informant interview with teachers, focus group discussions with students	High	No concerns related to methodology, relevance, coherence and adequate of data underlying the finding
26	The main barrier expressed by students was that the lessons were not examinable.	Key informant interviews with teachers and school leaders, focus group discussions with students	High	No concerns related to methodology, relevance, coherence and adequate of data underlying the finding
27	Teachers felt that teaching the lessons was an additional workload that was hard to manage.	Key informant interviews with teachers and school leaders	High	No concerns related to methodology, relevance, coherence and adequate of data underlying the finding
28	Competition for use of computers and projectors by other classes was a barrier to implementation of the intervention.	Key informant interviews with teachers and school leaders, focus group discussions with students	High	No concerns related to methodology, relevance, coherence and adequate of data underlying the finding

No	**Main findings**	**Methods and data sources underlying a finding**	**Overall CERQual assessment**	**Comment on the study finding**
29	The allocated time (40 minutes) was inadequate for teaching some lessons and competing priorities and time constraints were a major barrier to effective delivery of the intervention.	Key informant interviews with teachers and school leaders, focus group discussions with students	High	No concerns related to methodology, relevance, coherence and adequate of data underlying the finding
	**Factors facilitating scale-up of the intervention**			
30	The resources addressed skills that are important for students, teachers, and the public.	Key informant interviews with teachers, school leaders, and policymakers, focus group discussions with students	High	No concerns related to methodology, relevance, coherence and adequate of data underlying the finding
31	The lessons are compatible with the curriculum and integrating the lessons in specific subjects could facilitate scale-up of the intervention.	Key informant interviews with teachers, school leaders, and policymakers, focus group discussions with students	High	No concerns related to methodology, relevance, coherence and adequate of data underlying the finding
32	Making the content examinable would facilitate scale-up of the intervention.	Key informant interviews with teachers and school leaders, focus group discussions with students	High	No concerns related to methodology, relevance, coherence and adequate of data underlying the finding
33	Policy makers, teachers and students suggested the use of school clubs, printing of IHC resources and use of social media to scale up the IHC lessons.	Key informant interviews with teachers, school leaders, and policymakers, focus group discussions with students.	Low	There were concerns related to coherence and adequate of data underlying the finding
